# Ecological study on alien *Amaranthus spinosus* L. in the Egyptian Nile Valley

**DOI:** 10.1038/s41598-026-49216-5

**Published:** 2026-04-30

**Authors:** Fatma A. Ayed, Dalia Abd El-Azeem Ahmed

**Affiliations:** 1 Botany and Microbiology Department, Faculty of Science, Aswan University, Aswan, 81528 Egypt; 2 Botany and Microbiology Department, Faculty of Science, Tanta University, Tanta, 31527 Egypt

**Keywords:** *Amaranthus spinosus* L., Flora, Introduced species. Non-native, Noxious weed, Ecology, Ecology, Plant sciences

## Abstract

*Amaranthus spinosus* L. is a member of the Amaranthaceae family, commonly known as the spiny amaranth, or spiny pigweed. It is native to the tropical Americas, but is present on most continents as an introduced species, noxious weed, and can be a serious weed of rice cultivation in Asia. In Egypt, the Nile Delta, particularly Cairo, is the home of spiny amaranth; however, it has recently been recorded in the Nile Valley (Aswan area) colonizing different habitats such as roadsides, depressions, Khor and river banks. This study aimed to study the current distribution of spiny amaranth in the Aswan area and its associated species. It also aims at studying their life and growth forms, habitats, soil characteristics, local and global distributions, local threats, and spiny amaranth seed characteristics. Five sites along the river Nile in Aswan city was surveyed from the period of 2018 until 2025 in four identified habitats (Roadsides, depressions, khor, and river banks). Forty species were recorded associated with spiny amaranth, belonging to 38 genera and 16 families. Therophytes (15 species) followed by phanerophytes (14 species) were the most represented life form. All recorded species inhabit the Nile region, and most of the 12 taxa were pantropical. Most studied species (32.5% of the total threatened species) suffer from at least one type of threat. They mostly suffer from habitat loss due to industrial, urban, and tourist development. Soil samples were analyzed for edaphic variables. The relative importance of a certain environmental factor on the distribution of the recorded species across the four habitats were performed using PCORD (v6.22 software). The spiny amaranth seed colors are mainly dark brown to reddish brown, lenticular shape, glabrous and matte surface, acute apex, and obtuse to rounded base, hilum marginal, sub-basal, and slightly eccentric. Its length ranged from 7.24 to 7.84 mm, and its width varied between 7.27 and 7.77 mm. The authors recommended that monitoring the behavior of alien species and conducting ongoing surveys are crucial for biodiversity conservation.

**Supplementary Information:**

The online version contains supplementary material available at 10.1038/s41598-026-49216-5.

## Introduction

*Amaranthus spinosus* L. is a member of the Amaranthaceae family. It is commonly known as spiny amaranth, spiny pigweed, thorny amaranth, **or** prickly amaranth^1^. *Amaranthus* comes from the Greek word amarantus, which means “eternal.”^2^. It is a spiny, erect annual or perennial herb, up to 100–130 cm tall, heavily branched, monoecious herb with a purplish or greenish stem^3,4^. The leaves are alternating and simple, with no stipules; the petiole is about the same length as the leaf blade; the blade form is ovate-lanceolate to rhomboid^5^. Leaf petiole ± equaling or longer than blade; blade rhombic-ovate, ovate, or ovate-lanceolate, 3–10(−15) × 1.5–6 cm, base broadly cuneate, margins entire, plane or slightly undulate, apex acute or sub obtuse to indistinctly emarginate, mucronulate. Its stems and leaves are hairless and can sometimes have a reddish tinge. The fruit is dehiscent, compressed, ellipsoidal, acute or obtuse, with a short, inflated neck below the style base, circumsessile a little below the middle, or indehiscent. The fruits of most *Amaranthus* species are dark or brownish-black, except for *A. spinosus* L., which has light brown or brownish-yellow fruits^6^.

Inflorescences are simple or compound terminal staminate spikes and axillary sub-globose mostly pistillate clusters, erect or with reflexed or nodding tips, usually green to silvery green. Seed heads can be seen at the terminals of stems and in tiny clusters at leaf axils^7^. Male flowers occur on branches at the top of the plant, and female flowers occur on branches at the middle and bottom of the plant^8^. Bracts of pistillate flowers are lanceolate to ovate-lanceolate, shorter than tepals, apex is attenuate. Pistillate flowers: tepals 5, obovate-lanceolate or spatulate-lanceolate, equal or subequal, 1.2–2 mm, apex mucronate or short-aristate; styles erect or spreading; stigmas 3. Staminate flowers: often terminal or in proximal glomerules; tepals 5, equal or subequal, 1.7–2.5 mm; stamens 5. Utricles ovoid to sub-globose, 1.5–2.5 mm, membranaceous proximally, wrinkled and spongy or inflated distally, irregularly dehiscent or indehiscent (Committee^9^,.

The number of flowers 1 cm² is from 30 to 45^6^. Cotyledons are long, narrow, and glabrous. *Amaranthus spinosus* L. emerges in June, flowers in July, and the fruit develops in August, followed by seed dispersal in September. The seed has a diameter of about 1 mm, is glossy and compacted, and is black or brownish-black in colour^10^. The morphological characteristics of spiny amaranth are unique and influenced by plant adaptation and genetic variation (Fatinah et al., 2012).

Spiny amaranth is an *Amaranthus* species, a genus that now has the second-highest number of invasive alien plants (IAPs) (14 species) in China^11^; Yan et al., 2014). It can be adjusted to a wide range of Eco geographic and edaphic factors (environmental heterogeneity)^12^. The United States government has listed several plants as noxious weeds under the Plant Protection Act (7 U.S.C. 7701 et seq., 2000). Some of these plants are weedy or invasive, while others are prized natives in other parts of the country^1^. In China Spiny amaranth is one of the most toxic and aggressive weed species globally. It is classified as an intermediate invasive plant. The risk index value is 59, and China’s threat level is third^13^. These weeds are economically significant because they cause substantial yield losses in various field crops, vegetables, and orchards^14,15^. This species has not yet been assessed in the International Union for Conservation of Nature (IUCN) Red List of Threatened Species^16^.

It is widely used worldwide for its nutritional and therapeutic characteristics (as described in traditional medical systems) in treating a variety of ailments^17^. It is used as an antiviral, antimalarial, antidiabetic, antibacterial, antihelminthic, and snake antidote in traditional medicine^18–20^. It is widely cultivated in Kigali, Rwanda, due to its nutritional relevance and demand, and it is less expensive^21^.

It is a green leafy vegetable that is low in cost, easy to prepare, and high in nutrients such as vitamins, proteins, and minerals. Gupta and Wagle^22^,. It’s high in minerals, including manganese, iron, calcium, copper, magnesium, phosphorus, and potassium^23^. According to the original studies, individual risk factors, particularly those associated with cardiovascular disease, could be reduced by eating enough sodium, potassium, magnesium, calcium, manganese, copper, zinc, and iodine in amaranth. Regular amaranth consumption can help lower cholesterol levels and lower blood pressure^24^. Spiny amaranth has a carbohydrate content of 1.16 g/100 g leaves and a protein content of 4 g. The plant has a calcium value of 968.7 mg/100 g dry weight^25^.

It is thought to have originated in lowland tropical South and Central America and was introduced to other warmer parts of the world around 1700 AD. It grows gregariously and as a weed in tropical and subtropical climates, including tropical Africa. It is sometimes found in temperate zones as well. It is rarely cultivated^26^. In Egypt, it was classified as an introduced naturalized species, not as an invasive one, as it was a non-native plant that had successfully established self-sustaining, reproducing populations in a new environment without needing continued human intervention. They differ from invasive species by typically integrating into the ecosystem rather than causing widespread ecological harm^27^. Egypt’s Nile Delta, particularly Cairo, is home to it^28,29^.

This paper’s primary goal is to provide light on the autecology of *Amaranthus spinosus* in the Aswan area and extend to study and report its associated species and their life forms, growth forms, Local and global distribution, the anthropogenic effects, habitats, soil characteristics, and spiny amaranth seed characteristics.

## Materials and methods

### Study area

**Fig. 1:**
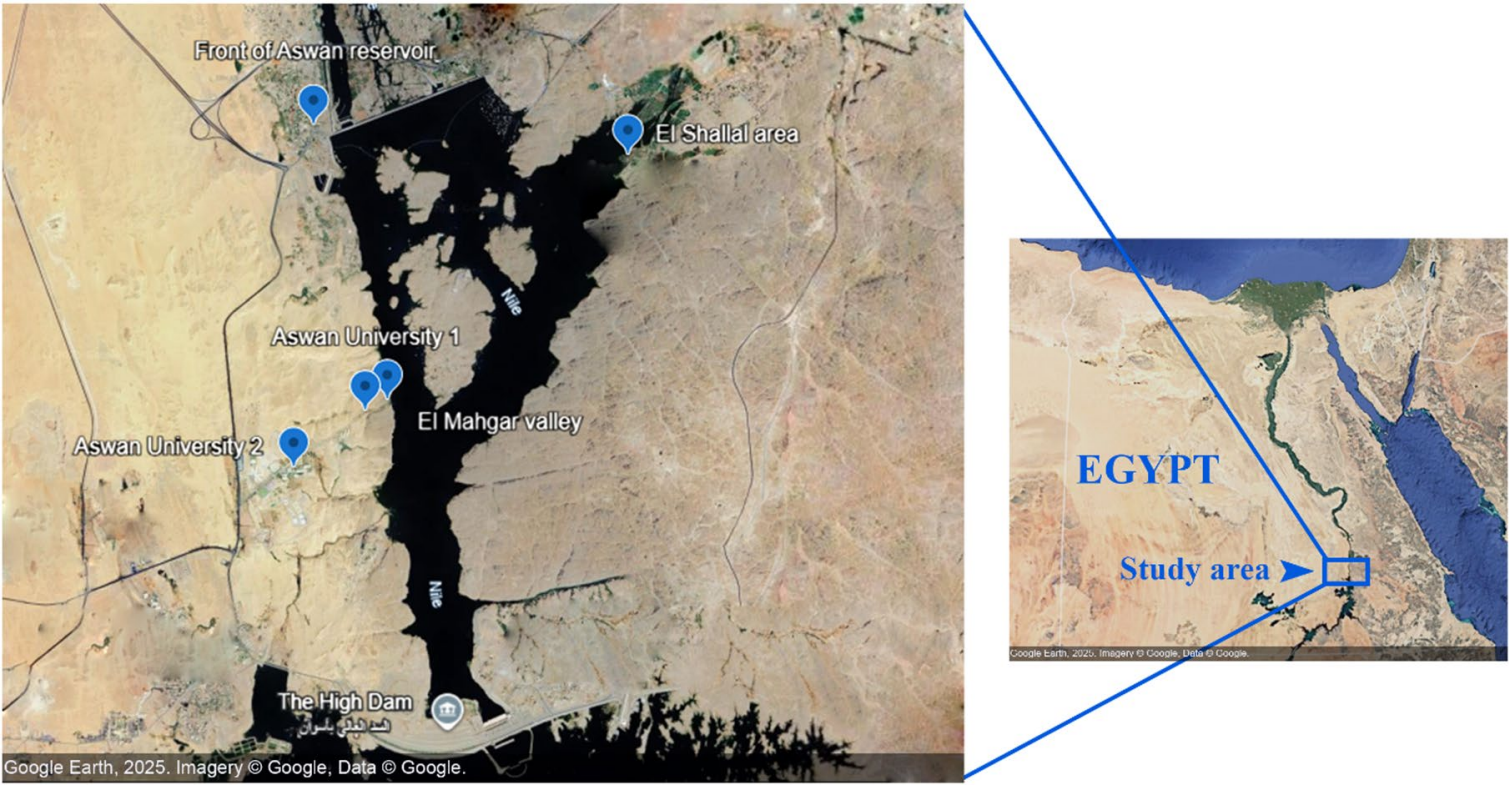
Map showing the study area along the Nile valley (Aswan city) with sampling sites. (Source: Google Earth, 2025. Imagery © Google, Data © Google).

The research was performed in the River Nile vegetation of Aswan city at five sites (Aswan university camps1and 2 sites, front of Aswan reservoir, El Shallal area, El Mahger valley) (Table 1; Fig. 1), which represent a distinctive arid-hydro environment characterized by the combined effects of shoreline vegetation zonation and high aridity. These locations did not include any protected or restricted areas, and all locations were publicly accessible. The study area covers for area in Aswan Governorate, Aswan, which lies between South Sudan at latitude 22 north of the tropic of Cancer and Luxor Governorate to the north. Aswan is located on the eastern shore of the Nile; it rises about 85 m above sea level and is 880 Km away from Cairo. It covers an area of 62.766 Km².

**Table 1:** Locations of the study area with their coordinates, Habitats, Human presence and Current human activities.

Sites	Latitude and longitude	Habitats	Human presence	Current human activities
Aswan University1	24° 00’ 14” 32° 52’ 05”	Roadsides	Inhabited	Aswan university
Aswan University 2	23° 59’ 53” 32° 51’ 36”	Depressions	Uninhabited	None
Front of the Aswan reservoir	24° 02’ 00” 32° 51’ 44”	Roadsides	Inhabited	Military checkpoint station
El Shallal area	24° 01’ 49” 32° 53’ 52”	khor	Uninhabited, regularly visited	Agricultural, fishing and tourism
El Mahgar Valley	24° 00’18” 32° 52’ 14”	Riverbank	Uninhabited, regularly visited	Fishing

**Fig. 2:**
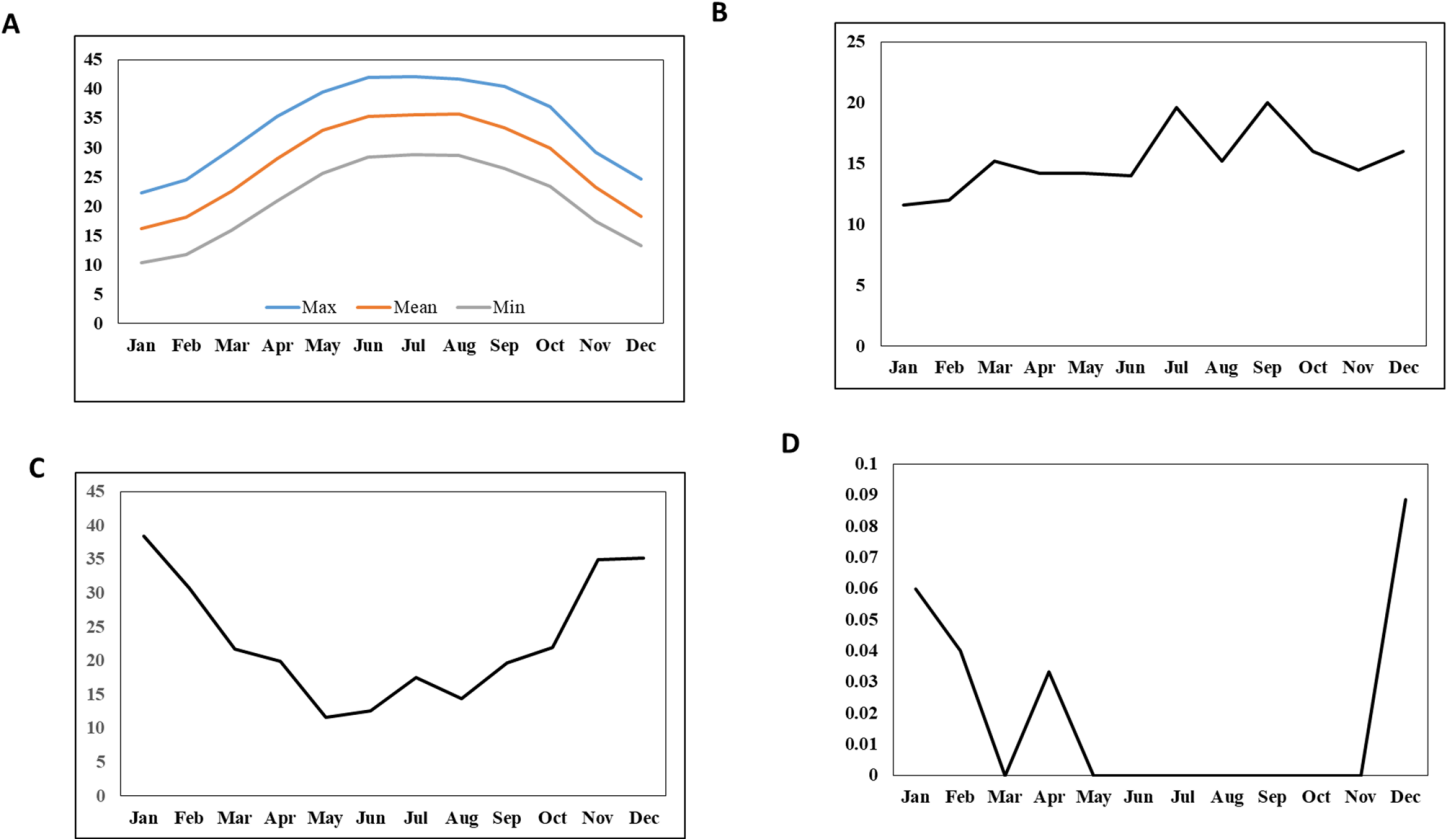
The monthly mean of the climatic parameters (**a**) temperature (°C); (**b**) wind speed (m/s); (**c**) relative humidity (%) and (**d**) precipitation (mm) for the study area averaged over the period from 2019 to 2023 (https://www.britannica.com/place/Nile-River/Climate-and-hydrology).

Aswan is mainly characterized by arid habitats, with most of the region occupied by the granites and sedimentary rocks. Rain is rare, randomly precipitated, and varies in amount throughout the year. Rainfall occurs in the cold season from November to May, but summer is practically rainless (Fig. 2). The area experiences a hot desert climate typical of Egypt, characterized by scorching summers with average high temperatures consistently exceeding 50 °C (https://www.britannica.com/place/Nile-River/Climate-and-hydrology).

## Field surveys and data collection

Successive field surveys for exploring the occurrence of the species and collections of associated plant material were conducted at different periods, from spring 2018 until spring 2025. Seasonal field trips were conducted to ensure the collection of plant material at different phenological stages, especially at flowering and fruiting stages. The collection was carried out in accordance with both national and local laws. There were no protected or endangered plant species involved, and the plants were collected ethically and without discrimination. To reflect the variety of ecosystems found in the study area. A total of 50 sampling plots, each measuring 1 × 1 m within 10 stands, were chosen at random to guarantee representation of habitat differences^30,31^. Floristic records were carried out, based on the presence and absence of species^32^ in the field. Habitats of the recorded species were identified following the study of Ayed^33^. In each site, the taxonomical identification and nomenclature for the recorded taxa were according to Täckholm^29^ and Boulos^34–36^, 2005^28^,. Updated according to Plants of the World online (https://powo.science.kew.org/*)* (POWO 2025) and World Flora Online (https://wfoplantlist.org/*).* The collected specimens of spiny amaranth underwent processing to create herbarium specimens, subsequently identified with Mohamed O. Badry^32^, and deposited at the Herbarium of Aswan University (ASW) and Tanta University Herbarium (TANE), with *Acc. No. 14,308*.

Life forms of the recorded species were identified following the system of Raunkiaer^37^, and the growth form of species was detected according to^18,38–40^. Local distribution of the recorded species was determined from the following reference^29,41,42^. The national geographical regions of the recorded species were coded as follows: Nd = Nile Delta, NV = Nile Valley, NF = Nile Faiyum, O = Oases of the Western Desert, Mw = Western Mediterranean coastal region, ME = Eastern Mediterranean coastal region, Da = Eastern desert, DI = Isthmic desert, Dl = Libyan Desert, R = Red Sea coastal region, GE = Gebel Elba and surrounding mountains and S = Sinai proper. Global distribution of the recorded species was collected from Zohary^43,44^, Feinbrun-Dothan^45^ and 1986), Shaltout and Al-Sodany (2002) and Seif El-Nasr and Bidak^46^ and Ayed^33^. The global distribution (i.e. floristic regions) was coded as follows: Cosm: Cosmopolitan, ME: Mediterranean, IR – TR = Irano–Turanian, SA – AR = Saharo–Arabian, ER – SR = Euro–Siberian, SU – ZA = Sudano–Zambezian, PAL = Palaeotropical, PAN = Pantropical, and Aus = Australian.

Threats encompass both direct and indirect factors leading to ecosystem degradation and species decline. Data on the type of threat influencing habitats and the recorded species were based on field observation and previous studies conducted on the investigated area (e.g^32,47–50^.,; Yassin, 2024^33^;. Six types of threat were identified: 1- over-collecting and over-cutting, 2- habitat loss due to industrial, urban, and touristic development, 3- browsing and overgrazing, 4- clearance for agriculture, 5- disturbance by cars or trampling, and 6- climatic changes and environmental conditions.

## Seeds characteristics

Mature seeds of Spiny amaranth were collected from the studied localities of the study area in September 2024. Seeds were separated from the dehisced seledpod (follicle) without any mechanical tool. Collected seeds were assessed for macro and micro seed morphology, including seed surface, color, hilum position, shape, and dimension, while quantitative characters evaluated were seed length/width ratio, and coma length/width ratio using Olympus SZ61 microscope^51,52^ with Toup camera. Measurements were based on (*n* = 5) mature seeds and expressed as mean ± standard deviation (SD).

## Soil analysis at each site

A composite soil sample was obtained from each site, consisting of three profiles per site, extending from 0 to 50 cm in depth. The soil samples were transferred to the laboratory and air-dried after being spread on sheets of paper. The gravel content of each sample was then eliminated by passing it through a 2 mm sieve. After estimating and discarding the gravel content, the remaining soil was saved for mechanical and chemical investigation. Twenty grams of dirt and one hundred milliliters of distilled water were shaken for an hour to create a soil extract (1: 5). The mixture was filtered using a Whatman No. 1 filter paper to obtain a clear filtrate. The filtrate was used in the subsequent analysis. Soil pH was determined in a 1:5 soil extract using an electric pH meter (Ohaus pH meter model ST2100, USA) with a glass electrode. Soil electrical conductivity (EC) was also measured in the soil filtrate using a conductivity meter (model ST300C conductivity/TDS meter, Ohaus Corporation, USA). The concentrations of soil cations, including sodium (Na), potassium (K), calcium (Ca), and magnesium (Mg), were assessed. Na and K concentrations were determined by flame photometry -model 400 flame photometer, Corning, UK^53^, while Ca and Mg concentrations were determined using the Versenate method^54^. Common soil anions, including chlorides, phosphates, and nitrates, were also assessed. Chlorides were volumetrically determined by titration against silver nitrate solution using potassium chromate as an indicator^55^. Dissolved inorganic orthophosphates were determined colorimetrically using phospho-molybdate according to^56^. Nitrate was determined spectrophotometrically using sodium salicylate in measuring absorbance at 420 nm according to^57^. The carbonates and bicarbonates were estimated by titration against 0.01 N HCl in the presence of methyl orange and phenolphthalein as indicators. A 5 ml of soil extract to which a few drops of phenolphthalein indicator were added, was titrated against 0.01 N HCl till orange colour appears, then drops of methyl orange were added to 5 ml of soil extract and the titration continued. The endpoint of phenolphthalein is equivalent to half of the carbonates, and the endpoint of methyl orange is equivalent to total alkalinity-carbonate and bicarbonate^58^. Soil texture was determined through mechanical analysis using the pipette method^59^, which separates different-sized particles. The percentages of sand, silt, clay, and gravel were calculated accordingly^60^. Organic matter content was determined using the loss-on-ignition method, where oven-dried soil samples were ignited at 450 °C for 24 h, and the loss in sample weight was used to determine organic matter percentage^61^.

### Data analysis

The relationship between vegetation habitats and environmental variation was assessed using Canonical Correspondence Analysis (CCA) using the software PC-ORD Multivariate Analysis of Ecological Data (Version 6.22; MjM Software Design, USA (http://www.pcord.com›)^62^. Additionally, the variation in the species diversity and soil variables in relation to vegetation habitats was assessed using one-way analysis of variance (ANOVA). All statistical analyses were conducted using Minitab 19.1^63^.

### Ethical considerations

The University of Aswan’s Faculty of Science Research Ethics Committee received the research protocol along with the plants and seeds that were gathered. Following the same institutional and regulatory norms, the Committee examined the study and authorized the exemption from ethical review. This study did not involve protected or endangered plant species. The protocol was designed and conducted in accordance with the Egyptian Government’s Ethical Guidelines and the Supreme Council of University Research Ethics Regulation.

## Result and discussion

### Floral results

A total of 40 species were recorded alongside *A*. *spinosus* in four different habitats (roadsides, depressions or slopes, Khor and river banks) during our study in the Nile valley, belonging to 38 genera and 16 families. The most species-rich families were Asteraceae (7 taxa = 17.5%), Fabaceae (6 taxa = 15%), and Poaceae (4 taxa = 10%). Among other families, Asteraceae, Fabaceae, and Poaceae are the most widely distributed in the studied region^32,64^. According to Shaheen^65^ and Abd El-Ghani and Fawzy^66^, this dominance is seen throughout the Egyptian environment and is not specific to the research location. Additionally, it was stated that these three families were the most prevalent in North Africa, the Mediterranean, northern Zambia, and eastern Ethiopia^67^. Their diverse biological range of tolerance and seed dispersal efficacy across local conditions of water depth may be the cause of this spread^68^.

**Fig. 3:**
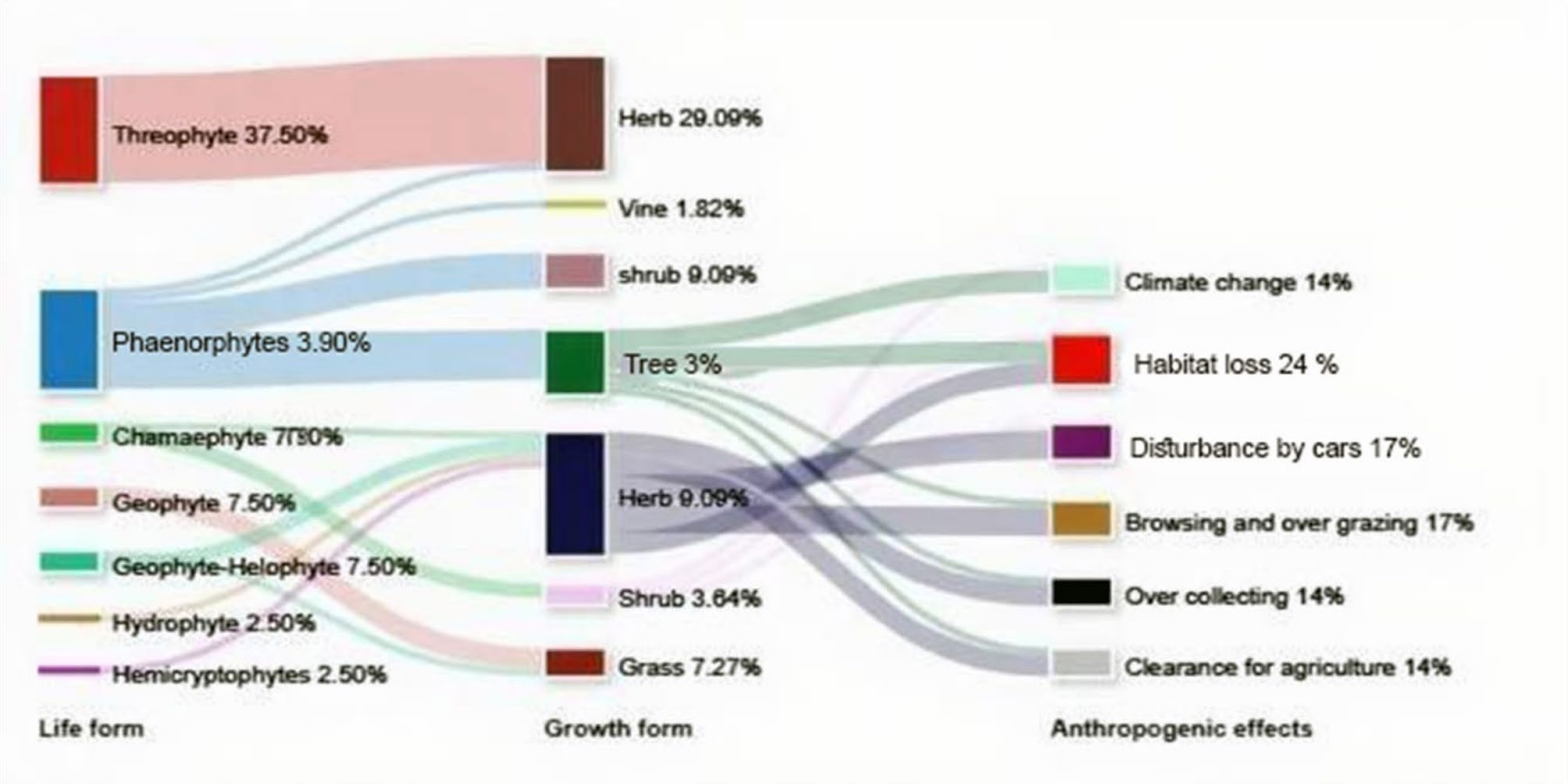
Sankey diagram illustrating linkages between life form, Growth form, and anthropogenic effects of *Amranthus spinosus* L. associated plants in the Egyptian Nile Valley (Aswan city).

The determination of the life forms (Table S1) of the recorded species indicated that the therophytes are the most represented (15 taxa = 37.5% of the total species), followed by phanerophytes (14 taxa = 35%), geophytes-helophytes (3 taxa = 7.9%), geophytes (3 taxa = 7.9%), chamaephytes (3 taxa = 7.9%), hemicryptophytes and helophytes (1 species = 2.5%) for each one. These results are similar to those reported by^64,69^. The dominance of therophytes is attributed to severe climatic conditions, moisture scarcity, substrate instability, and biotic effects^70^. Most of the growth form species (21 taxa) were herbs, followed by shrubs (7 taxa), trees (7 taxa), and grass represented (4 taxa), while vines were represented by only one taxon (Fig. 3). Most of the growth form species in the study area were herbs, which may be related to the human impact and cultivated islands. This postulation was supported by^33,71^.

### Local and global distribution

National geographical distribution showed that six taxa (15%) occur in only one phytogeographical region. In addition, 5 taxa occur in 2 regions (12.5%), and 7 taxa (17.5%) had a wide geographical distribution all over Egypt (occur in all the 12 regions). *Cynodon dactylon*, *Cyperus rotundus*, *Echinochloa colona*, *Phoenix dactylifera*, *Pulicaria undulate*, *Solanum nigrum* and *Tamarix nilotica* (Table S1). The Nile region is the richest region with species, comprising 40 taxa (100%) of the total species, followed by the Mediterranean and Oases region (22 taxa each = 55%), the Desert by 21 species (52.5%), and the Gebel Elba region has the least number of species (12 taxa = 30%). The Nile Region (40 taxa) has the greatest number of species in the current study. This great species richness could be explained by the diversity of the habitats found there, which span much of the environmental gradient from terraces to open water bodies. This lends credence to the idea that species diversity within habitats is enhanced by increasing habitat heterogeneity^72^. It contributes about 29.9% of the total flora of Egypt^73^.

The global distribution (floristic region) of the plant species showed that the majority of the recorded flora (12 taxa = 30% of all recorded taxa) were Pantropical, followed by Cosmopolitan (5 taxa = 12.5% of all recorded taxa), and Palaeotropical (4 taxa = 10% of all recorded taxa) (Table S1). The most prevalent chorotypes in the Nile islands region were pantropical, cosmopolitan, and palaeotropical, according to studies by Hamada^49^, Hamed et al.^69^,, Abd El-Ghani et al.^71^,, and Amer et al.^74^,. This suggests that the investigated area is more vulnerable to human disturbances due to its simpler floristic structure compared to other places in Egypt^75^.

### Anthropogenic effects (Local Threats)

Thirteen species (32.5% of the total threatened species) suffer from at least one type of threat (Table S1). The most common concern is habitat loss due to industrial, urban, and tourist development. Globally, irrational human activity is always endangering ecological integrity^76^. There has been a discernible decrease in biodiversity during the last century. Up to 30% of all species may go extinct by 2050, according to predictions made in 2007^77^. Loss of habitat, poor land management, excessive collection, excessive grazing, and climate change are the well-established risks^78^. One of the biggest threats to many species of Egyptian flora is habitat loss^79^. As a result, these areas have recently seen a number of difficulties, including an increase in tourism. In addition to destroying the habitats, this has caused the large tracts of habitat that surround them to deteriorate^80^.

### Habitats and soil characteristics

**Fig. 4:**
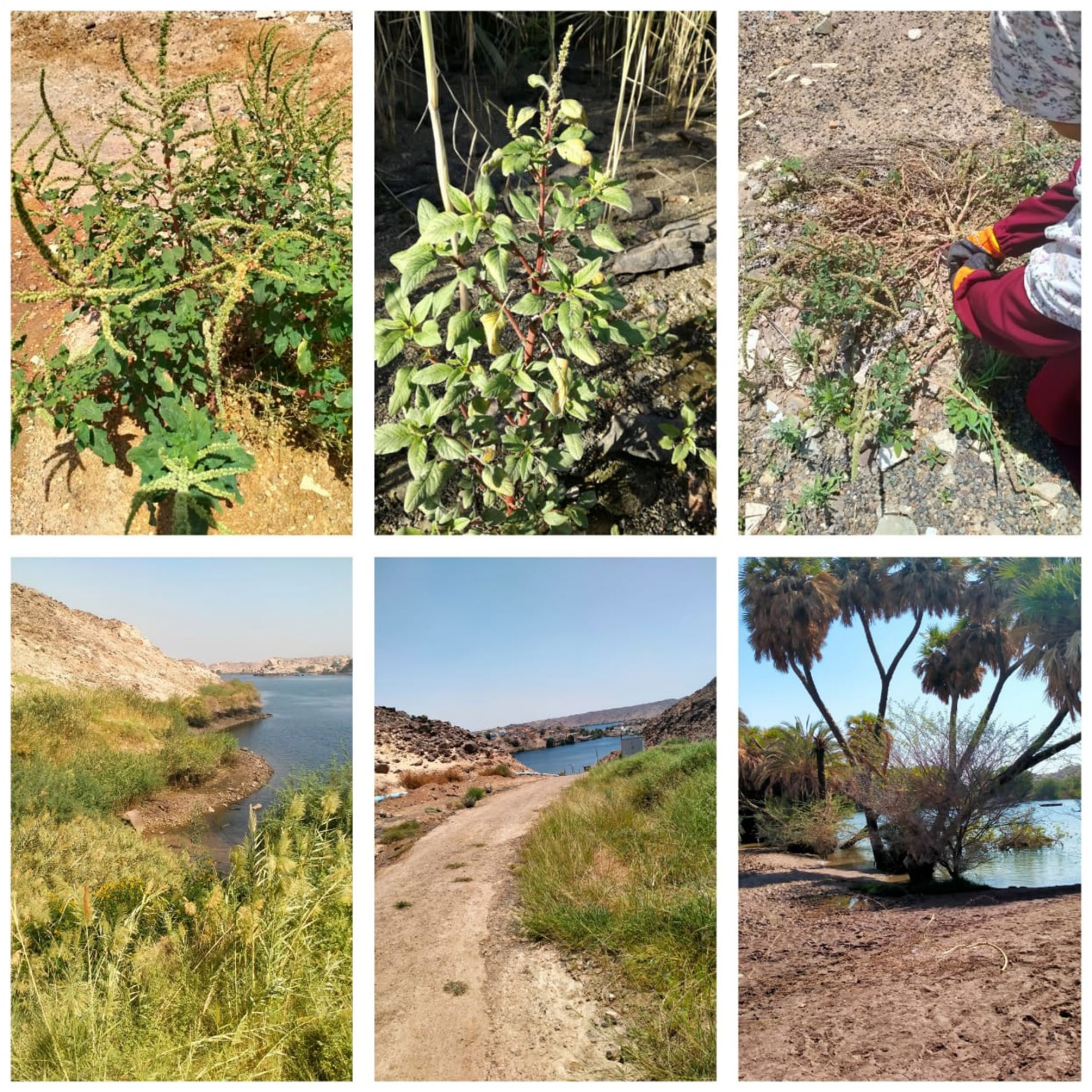
Field photography and habitats of *A. spinosus* were recorded in the Nile valley: Aswan city (taken by the authors).

Spiny amaranth can grow in a variety of environments. In our study, it can be preferred in areas such as roadsides, depressions, Khor, and river banks (Fig. 4). It grows in both wet and dry environments but thrives when soil moisture levels are below field capacity. Its growth is slowed by waterlogging. Soils with a high organic matter content, a loamy texture, and sufficient nitrogen yield the best results (Holm et al.,1977). Pigweed is a common weed in fields, pastures, orchards, disturbed ground, along roadsides, and in secondary forests^81^. On the Galapagos Islands, it grows in arid lowlands and damp uplands^82^. It thrives as an environmental weed in disturbed regions and rainforests, as well as an agricultural weed from near sea level to 820 m in Australia^83^. The species recorded in the different habitats with presence and absence at the five studied sites are shown in Table 2. Two species (*Leptadenia arborea* and *Calotropis procera*) have broad ecological amplitude and were recorded in all habitats^64^.

**Table 2:** Presence and absence of all recorded species in different habitats.

Species	Road sides	Depression	Khor	River banks
*Aerva javanica* (Burm.f.) Juss. ex Schult.		+		
*Ageratum conyzoides* L.				+
*Alternanthera sessilis* (L.) Dc.				+
*Amaranthus spinosus* L.	+	+	+	+
*Aster squamatus* (Spreng.) Hieron			+	+
*Blumea bovei* (DC.) Vatke		+		
*Calotropis procera* (Aiton) Dryand.	+	+		
*Coronopus didymus* (L.) Sm.				+
*Coronopus squamatus* (Forssk.) Asch.				+
*Cynodon dactylon* (L.) Pers				+
*Cyperus longus* L				+
*Cyperus rotundus* L.			+	+
*Dalbergia sissoo* Roxb. ex DC.	+			
*Dichanthium annulatum* (Forssk.) Stapf		+		
*Echinochloa colonum* (L.) Link				+
*Eucalyptus camaldulensis* Dehnh.			+	
*Glinus lotoides* L.			+	
*Hyphaene thebaica* (L.) Mart.			+	
*Lactuca serriola* L.				+
*Lantana camara* L.	+			+
*Leptadenia arborea* (Forssk.) Schweinf.	+	+	+	+
*Leucaena leucocephala* (Lam.) de Wit	+			
*Phoenix dactylifera* L	+		+	
*Phragmites australis* (Cav.) Trin. ex Steud.		+		+
*Phyla nodiflora* (L.) Greene			+	+
*Physalis angulata* L.				+
*Pluchea dioscoridis* (L.) DC.		+	+	+
*Portulaca oleracea* L.				+
*Psidium guajava* L.				+
*Pulicaria undulata* (L.) C.A.Mey.		+		
*Rorippa palustris* Besser				+
*Rumex dentatus* L.		+		
*Salsola imbricata* Forssk.		+		
*Senna didymobotrya* (Fresen.) H.S.Irwin & Barneby				+
*Sesbania sesban* (L.) Merr		+	+	+
*Solanum nigrum* L.			+	
*Sonchus oleraceus* L.				+
*Tamarix senegalensis* DC.		+	+	
*Trigonella glabra* Thunb.				+
*Vachellia nilotica* subsp. *tomentosa* (Benth.) Kyal. & Boatwr.			+	
*Veronica anagallis-aquatica* L.		+		+

**Fig. 5:**
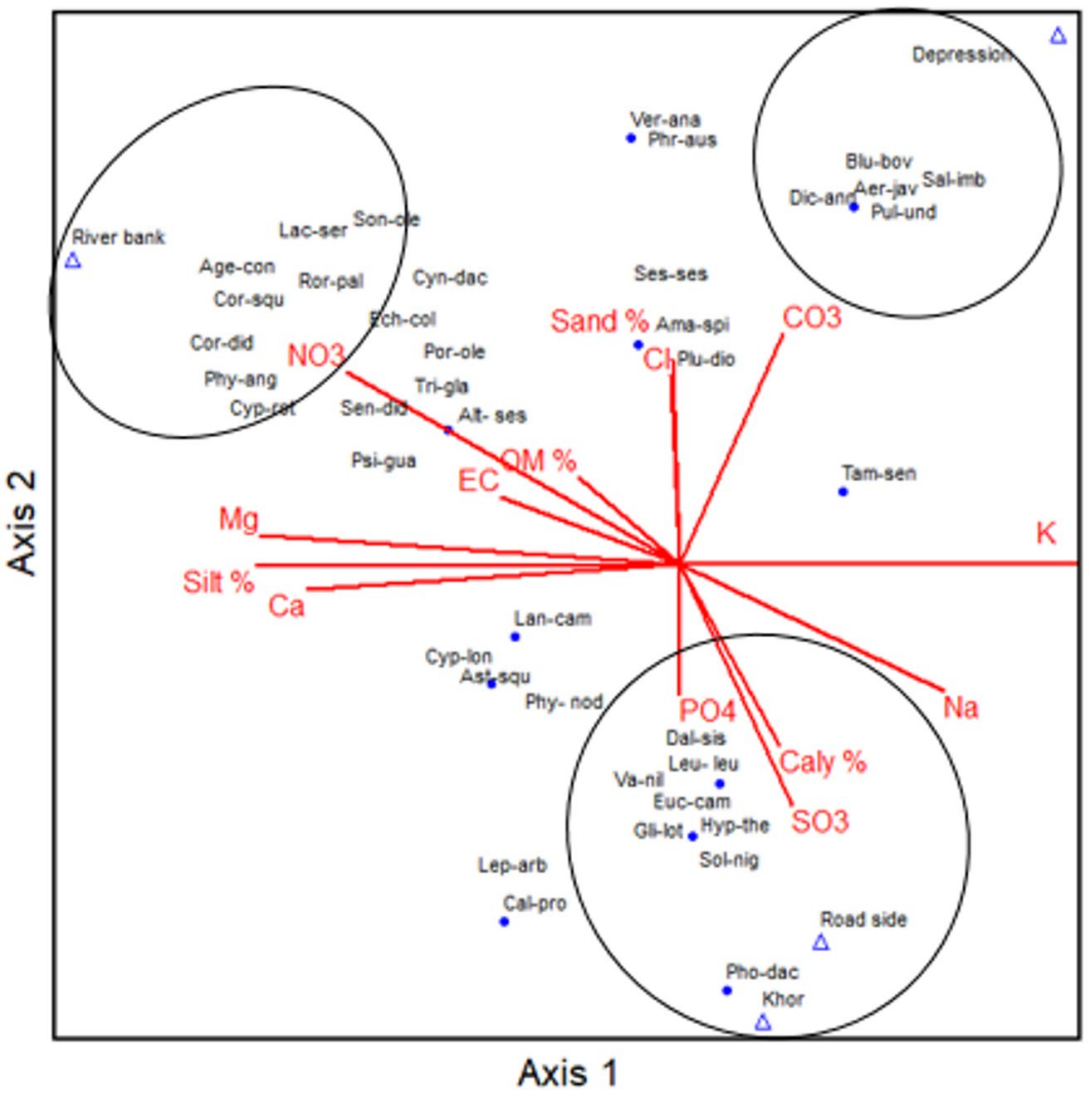
Canonical Correspondence Analysis (CCA) ordination biplot, showing the relationship between the environmental variables (represented by the red arrows) and recorded species across the four habitats. Species names are abbreviated with the first three letters of the genus and the first three letters of the species (e.g. *Aerva javanica* : Aer-jav).

The one-way ANOVA indicates that there are significant differences between the soil variables of the various habitats Table 3. PH values showed that most of the soils are alkaline and non-saline, where pH ranges from 7.9 in soils of roadsides habitats to 7.37 in soils of Khor habitats. EC reached the maximum value in soils of River banks habitats, while its minimum value was recorded in Road sides habitats. Soil samples are characterized by very low content of organic matter. It ranged from 0.63% in soils of Road sides to 1.04% in soils of River banks.The soil of Depressions had the highest contents of sands (82.67%) and CO_3_^2−^ (2.56 mg/g), but the lowest contents of silt (4.53%). The soil of Road sides had the highest content of clay (18.37%) and Na^+^ (29.9 mg/g), but the lowest Ca^2+^ (0.56 mg/g), Mg^2+^ (0.34 mg/g). The soil of khor had the highest PO_4_^2−^ (0.048 mg/g), but the lowest Cl ^−^ (0.063 mg/g). While the soil of River banks had the highest Ca^2+^ (0.624 mg/g), and the lowest Clay (9.64%). The CCA ordination biplot provides insight on the pattern of plant species distribution and relates it to environmental variables (Fig. 5). The arrow length of environmental variables along the CANOCO ordination biplot (Fig. 18). Expresses the relative importance of a certain environmental on the distribution of the recorded species across the four habitats. The most important edaphic factor in the distribution of the four habitats species are the sand, clay and silt, NO_3_^2−^,Mg, Ca, Na, CO_3_^2−^contents; while other soil variables seem less effective in controlling the distribution of the species. PC-ORD is essential for converting intricate ecological data into comprehensible representations such as dendrograms and ordination graphs. They offer some of the most clear and efficient methods for comprehending complex community patterns^84^. The important soil gradients related to the distribution of vegetation as recognized by^85,86^ are soil salinity (EC), moisture gradients, soil fertility (organic carbon, phosphorus, and nitrogen contents), soil texture (sand, silt and clay), pH value and calcium carbonate content. In the present study, the ordination technique indicated that sand, clay and silt, NO_3_^2−^, Mg, Ca, Na, CO_3_^2−^contents to the distribution of the four habitats species. While other soil factors appear to be less efficient in limiting the species’ spread.

**Table 3:** Soil characteristics of the 4 habitat types identified in Nile valley. (mean ± standard deviations).

Edaphic factors	Depressions	Road sides	khor	River banks
PH	7.43 ± 0.055	7.9 ± 0.3	7.37 ± 0.207	7.493 ± 0.159
EC (µs/cm)	122.5 ± 4.5	103.5 ± 1.5	124.3 ± 21	132.67 ± 2.89
Organic matter %	0.749 ± 0.258	0.637 ± 0.045	0.864 ± 0.134	1.04 ± 0.117
Sand %	82.67 ± 0.143	75.74 ± 0.27	81.22 ± 0.13	81.61 ± 0.03
Silt %	4.53 ± 0.133	5.31 ± 0.216	7.07 ± 1.27	8.76 ± 0.75
Clay %	11.58 ± 0.126	18.37 ± 0.24	12.1 ± 0.1	9.64 ± 0.24
Na^+^ (mg/g)	16.02 ± 5.85	29.9 ± 1.23	19.05 ± 5.18	9.71 ± 2.48
K^+^ (mg/g)	1.31 ± 0.566	1.779 ± 0.09	1.499 ± 0.382	0.631 ± 0.21
Ca^2+^ (mg/g)	0.6 ± 0.1	0.567 ± 0.167	0.622 ± 0.051	0.624 ± 0.181
Mg^2+^ (mg/g)	0.4 ± 0.08	0.34 ± 0.06	0.413 ± 0.046	0.427 ± 0.201
Cl ^−^ (mg/g)	0.095 ± 0	0.071 ± 0.024	0.063 ± 0.018	0.079 ± 0.027
CO32- (mg/g)	2.56 ± 0.019	1.62 ± 0.279	1.757 ± 0.434	2.078 ± 0.317
PO_4_^2−^ (mg/g)	0.014 ± 0	0.013 ± 0	0.048 ± 0.035	0.016 ± 0.003
NO_3_^−^ (mg/g)	0.19 ± 0.025	0.139 ± 0.0137	0.143 ± 0.016	0.196 ± 0.045
SO_4_^2−^ (mg/g)	0.091 ± 0.025	0.125 ± 0	0.167 ± 0.057	0.0527 ± 0.01

Hamada^49^, reported that sand and clay were effective edaphic factors in controlling the species distribution and vegetative grouping in the Nile valley. Calcium ions play an important role as a component of the carbonate system, as well as its apparent involvement in photosynthetic bicarbonate utilization (Ayed.2025). CO3 enhance the growth of weed communities associated with cultivated land^87,88^. The role of pH had negligible variation between study sites that had water with alkaline PH. A similar conclusion was reached by^89^.

### Seed characteristics

**Fig. 6:**
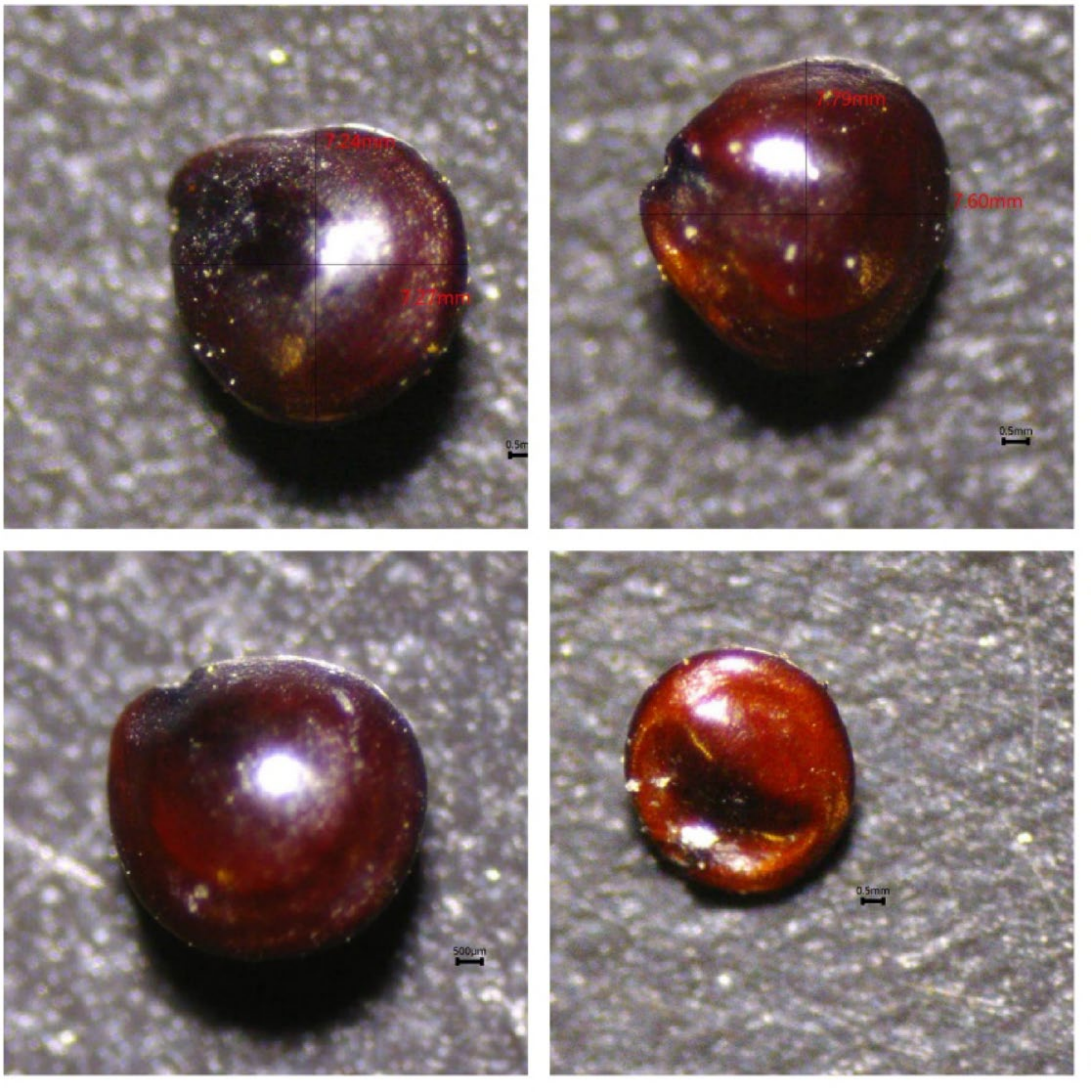
Seed characteristics of *Amaranthus spinosus* L. using Olympus SZ61 microscope with Toup camera (Taken by Author).

The qualitative and quantitative traits of the studied Spiny amaranth are shown in Table 4. The seed colors are mainly dark brown to reddish brown, lenticular shape, glabrous and matte surface, acute apex, and obtuse to rounded base, hilum marginal, sub-basal, and slightly eccentric (Fig. 6). The length of the seed ranged from 7.24 to 7.84 mm, with a mean value of 7.56 ± 0.25 mm. Seed width varied between 7.27 and 7.77 mm (mean = 7.58 ± 0.19 mm). The length/width (L/W) ratio was 0.99 ± 0.02. The morphology of seeds is a vital agricultural attribute, since it reflects a synthesis of genetic, physiological, and environmental factors, all of which profoundly influence crop production, quality, and market value^90^. Seed morphology has demonstrated utility in elucidating taxonomic connections within plant groups. Consequently, when evaluating plant biodiversity, seed size and shape are also important factors^51^. Spiny amaranth seeds stand out for their high gluten-free protein content and nutritionally balanced amino-acid composition^91,92^ and are a good source of palmitic, oleic, and linoleic acids^93^.

**Table 4:** Seed quantitative and qualitative characteristics of *Amaranthus spinosus* (Mean ± SD) recorded in the Egyptian Nile Valley (Aswan City).

Character	Description
Qualitative	
Size	Very small
Shap**e**	Lenticular, suborbicular
Seed surface	Glabrous, matte
Seed color	Dark brown to Reddish brown
Apex	Acute
Base	Obtuse to rounded
Hilum occurrence	Visible
Hilum position	Sub-basal, marginal
**Quantitative**	
Length (mm)	7.56 ± 0.25
Width (mm)	7.58 ± 0.19
Length/width	0.99 ± 0.02

### Origin and alien status category

**Fig. 7:**
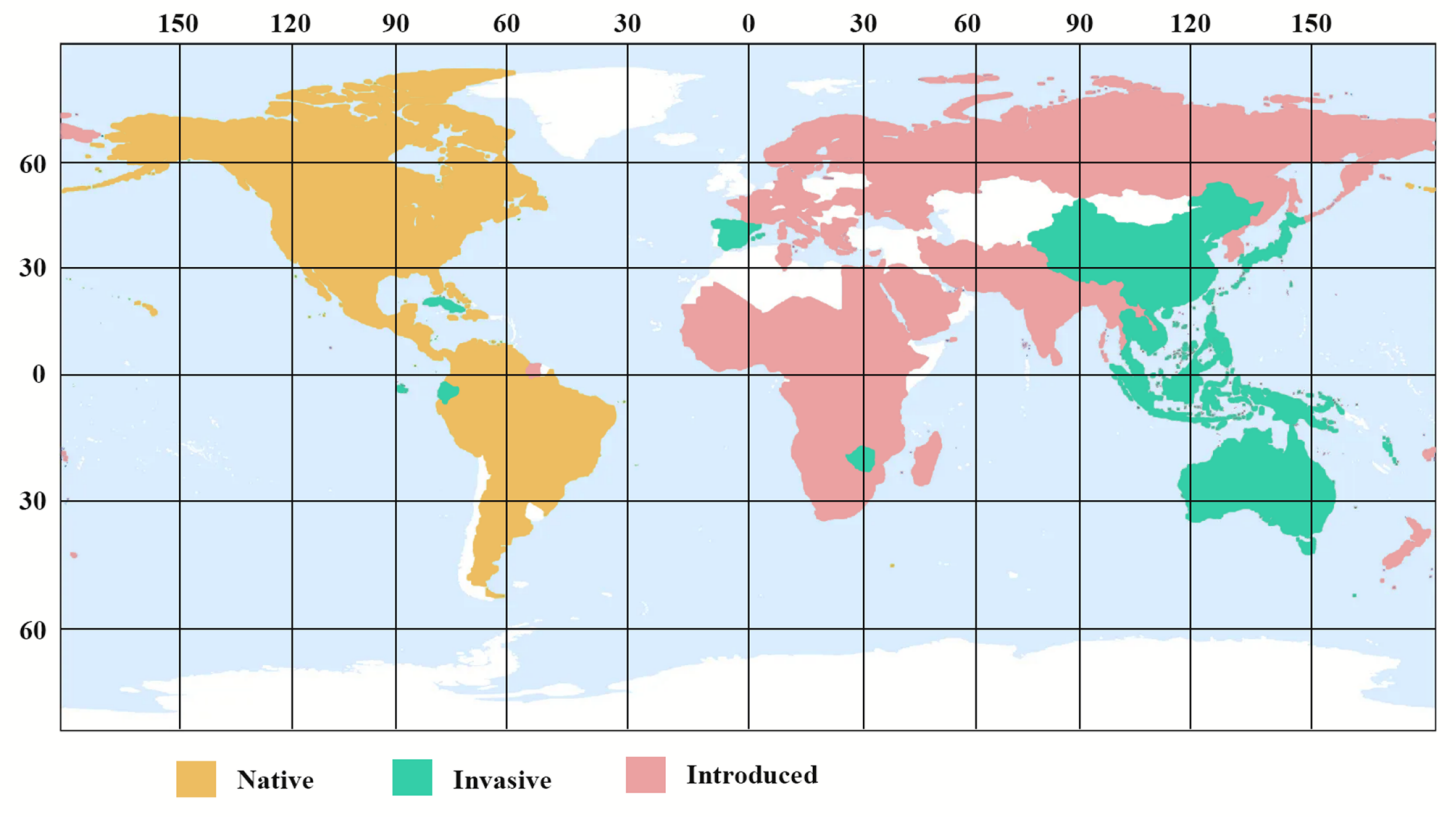
Native, invasive, and introduced *Amaranthus spinosus* L. all over the world (https://www.picturethisai.com/wiki/Amaranthus_spinosus.html*).*

*Amaranthus* is the largest genus in the Amaranth family (Amaranthaceae), with 70 species native to America, Africa, Australia, Asia, and Europe^94^. *Amaranthus spinosus* is a pantropical, widespread weed and is the only species in the genus armed with spines (Pal. 1972). It comes from the American tropics (Sauer 1967). The plant grows in tropical, subtropical, and Himalayan regions^3^, and is found in the lower to middle hills (3000–5000 feet) of the entire north eastern Himalayas^95,96^. Is found as a weed in both cultivated and fallow fields throughout Asia’s tropics and warm temperate zones, from Japan to Indonesia through India, the Pacific islands, and tropical America and Australia^97^. It’s a prevalent plant in Ghana’s wastelands, roadsides, and route sides, as well as along rivers, and it’s also found growing wild in Bangladesh^98^. *Amaranthus spinosus*, also known as prickly amaranth and regionally in Pakistan as Khaddar-chaulai, and in Hindi as “Kate Wali Chaulai (Kanatabhajii),” is a vegetable grown in India, Sri Lanka, and other tropical nations^99^. Records of *Amaranthus spinosus* on the North Atlantic plains of Morocco^100^. It is now regarded as an invasive plant in many tropical nations worldwide (e.g. Waterhouse. 1994;^101–112^(Fig. 7).

## Conclusion

*Amaranthus spinosus* L. is recorded as an introduced species to the Egyptian flora of the Nile Valley (Aswan area). Its first home was Cairo city, but now it has been recorded in another phytogeographical region (Nile Valley). The establishment of spontaneous populations in areas where the species has been introduced presents a potential risk. Similar to the early stages of invasion observed in other regions, there have been recent reports of the species in Egypt, especially in the Nile Valley, freshly reclaimed agricultural fields, and disturbed desert margins. This alignment suggests that Egypt may be approaching a critical ecological window where the species can change from an introduced component to an actively invasive component of the native flora. In order to prevent disturbance of biodiversity, the authors recommended ongoing monitoring of introduced species’ habits.

## Supplementary Information

Below is the link to the electronic supplementary material.

Supplementary Material 1

## Data Availability

The data that support the findings of this study are available from the corresponding author upon reasonable request.
