# Supplementary material for: Ecological study on alien Amaranthus spinosus L. in the Egyptian Nile Valley
Source: Sci Rep. 2026 Apr 30;16:13892. doi: 10.1038/s41598-026-49216-5 (PMC13133166; doi:10.1038/s41598-026-49216-5)
Supplement: Supplementary file 1 — Supplementary Material 1 [file 41598_2026_49216_MOESM1_ESM.docx]

**Supplementary materials**

**Table S1.** Accompanying Species of *Amaranthus spinosus* L., families, life form, Growth form, Global and national distribution, and anthropogenic effects (threats). Life form coded as: Ch: chamaephytes, Th: therophytes, Ph: phanerophytes, Ge-He: geophytes-helophytes, and He: helophytes. Global distribution coded as: Cosm: Cosmopolitan, ME: Mediterranean, IR – TR = Irano-Turanian, SA–AR = Saharo–Arabian, ER–SR = Euro–Siberian, SU–ZA = Sudano–Zambezian, PAL = Palaeotropical, PAN = Pantropical, and Aus = Australian. National distribution coded as: Nd = Nile Delta, Nv = Nile Valley, Nf = Nile Faiyum, O = Oases of the Western Desert, Mw = Western Mediterranean coastal region, Me = Eastern Mediterranean coastal region, De = Eastern desert, DI = Isthmic desert, Dl = Libyan Desert, R = Red Sea coastal region, GE = Gebel Elba and surrounding mountains and S = Sinai proper**.** And anthropogenic effects (threats) coded as: OC= Over-collecting and over-cutting, HL= Habitat loss due to industrial, urban, and touristic development, BO= Browsing and overgrazing, CA= Clearance for agriculture, DC=- Disturbance by cars or trampling, and CC= Climatic changes and environmental conditions.

| **Families** | **Species** | **Life form** | **Growth form** | **Global distribution** | **National distribution** | **Anthropogenic effects (Threats)** |
| --- | --- | --- | --- | --- | --- | --- |
| Amaranthaceae Juss. | *Aerva* *javanica* (Burm.f.) Juss. ex Schult. | Ch | Shrub | SU-ZA + SA-AR | Nv, De, Dl, R |  |
|  | *Alternanthera* *sessilis* (L.) Dc. | Th | Herb | PAN | Nd, Nv, De |  |
|  | *Amaranthus spinosus* L. | Th | shrub | PAN | Nv |  |
|  | *Salsola* *imbricata* Forssk. | Ch. | Shrub | SA-AR + IR-TR + SU-ZA | Nd, O, De, Dl, GE, S |  |
| Apocynaceae Juss. | *Calotropis* *procera* (Aiton) Dry and. | Ph | Shrub | SA-SI | Nv, Nf, O, DI, DL, R, GE, S |  |
|  | *Leptadenia arborea* (Forssk.) Schweinf. | Ph | vine | SU-ZA | Nv |  |
| Arecaceae Bercht. & J.Presl | *Hyphaene thebaica* (L.) Mart. | Ph | Tree | SA-ZU | Nv, Nf, De, S, R | CC |
|  | *Phoenix dactylifera* L | Ph | Tree | SU-ZA+SA-SI | Nd, Nv, Nf, O, MW, Me, De, DI, DL, R, GE, S | CC |
| Asteraceae Giseke | *Ageratum conyzoides* L. | Th | Herb | PAN | Nd, Nv, Nf , O |  |
|  | *Aster squamatus* (Spreng.) Hieron | Th | Herb | PAN | Nd, Nv, Nf, Mw, Me, De, DI, DL.GE |  |
|  | *Blumea bovei* (DC.) Vatke | Th | Herb | ME | Nv | OC, CA, DC |
|  | *Lactuca serriola* L. | Th | Herb | ME+IR-TR | Nd, Nv, O, Mw ,S |  |
|  | *Pluchea dioscoridis* (L.) DC. | Ph | Herb | SA-ZU+SA-SI | Nd, Nv, Nf, O, Mw, Me, De |  |
|  | *Pulicaria undulata* (L.) C.A.Mey. | Ch | Herb | SU-ZA+SA-SI | Nd, Nv, Nf, O, Mw, Me, De, DI, DL, R, GE, S |  |
|  | *Sonchus oleraceus* L. | Th | Herb | COSM | Nd, Nv, Nf, O, Mw, Me, De, DI, DL, R, S |  |
| Brassicaceae Burnett | *Coronopus didymus* (L.) Sm. | Th | Herb | ME + ER-SR + IR-TR | Nv |  |
|  | *Coronopus squamatus* (Forssk.) Asch. | Th | Herb | ME + ER-SR + IR-TR | Nd, Nv |  |
|  | *Rorippa palustris* Besser | Th | Herb | COSM | Nd, Nv, Mw, Me |  |
| Cyperaceae Juss. | *Cyperus longus* L | Ge - He | Herb | ME | Nd, Nv, Mw, Me | HL, BO, DC |
|  | *Cyperus rotundus* L. | Ge - He | Herb | PAN | Nd, Nv, Nf, O, Mw, Me, De, DI, DL, R, GE, S |  |
| Fabaceae Juss. | *Dalbergia sissoo* Roxb. ex DC. | Ph | Tree | PAN | Nv |  |
|  | *Leucaena leucocephala* (Lam.) de Wit | Ph | Tree | PAL | Nv |  |
|  | *Senna didymobotrya* (Fresen.) H.S.Irwin & Barneby | Ph | Shrub | PAN | Nd, Nv | CC |
|  | *Sesbania sesban* (L.) Merr | Ph | Shrub | SU-ZA | Nd, Nv, Nf, O, Me, R |  |
|  | *Trigonella glabra* Thunb. | Th | Herb | ME+SU-ZA+SA-SI | Nd, Nv, Nf, O, Me | HL, BO, CA, DC |
|  | *Vachellia nilotica* subsp. *tomentosa* (Benth.) Kyal. & Boatwr. | Ph | Tree | SU-ZA | Nd, Nv, Nf, O, DL, S | HL.BO |
| Molluginaceae Bartl. | *Glinus lotoides* L. | Th | Herb | PAL | Nd, Nv, Mw, Me, DI, DL, GE,S |  |
| Myrtaceae Juss. | *Eucalyptus camaldulensis* Dehnh. | Ph | Tree | AUS | Nv | OC, HL, CA, |
|  | *Psidium guajava* L. | Ph | Tree | PAN | Nd, Nv | HL.CC |
| Plantaginaceae | *Veronica anagallis-aquatica* L. | He | Herb | COSM | Nd, Nv, Nf, O, Mw, Me, DI, DL, GE, S |  |
| Poaceae Barnhart | *Cynodon dactylon* (L.) Pers | Ge | Grass | PAN | Nd, Nv, Nf, O, Mw, Me, De, DI, DL, R, GE, S |  |
|  | *Dichanthium annulatum* (Forssk.) Stapf | Ge | Grass | PAL | Nd, Nv, Nf, O, Mw, DI, R |  |
|  | *Echinochloa colonum* (L.) Link | Ge | Grass | PAN | Nd, Nv, Nf, O, Mw, Me, De, DI, DL, R, GE, S |  |
|  | *Phragmites australis* (Cav.) Trin. ex Steud. | Ge - He | Grass | PAL | Nd, Nv, Nf , O, Mw, Me, De, DI, DL, R, S |  |
| Polygonaceae Juss. | *Rumex dentatus* L. | Th | Herb | ME+IR-TR+SU-ZA | Nd, Nv, Nf, O, Mw, Me, | OC, BO, DC |
| Portulacaceae Juss. | *Portulaca oleracea* L. | Th | Herb | COSM | Nd, Nv, Nf, O, Mw, Me, R, S | OC, BO, CA |
| Solanaceae Juss. | *Physalis angulata* L. | Th | Herb | PAN | Nd, Nv | OC, HL |
|  | *Solanum nigrum* L. | Th | Herb | COSM | Nd, Nv, Nf, O, Mw, Me, De, DI, DL, R, GE, S |  |
| Tamaricaceae | *Tamarix senegalensis* DC. | Ph | Shrub | SU-ZA+SA-SI | Nd, Nv, Nf, O, Mw, Me, De, DI, DL, R, GE, S |  |
| Verbenaceae J.St.-Hil. | *Lantana camara* L. | Ph | Shrub | PAN | Nd, Nv |  |
|  | *Phyla nodiflora* (L.) Greene | Hemi | Herb | PAN | Nd, Nv, Nf, O, Mw, Me, De, DL | HL, BO, CA, DC |
